# Genome-scale metabolic reconstructions of *Pichia stipitis *and *Pichia pastoris *and *in silico *evaluation of their potentials

**DOI:** 10.1186/1752-0509-6-24

**Published:** 2012-04-04

**Authors:** Luis Caspeta, Saeed Shoaie, Rasmus Agren, Intawat Nookaew, Jens Nielsen

**Affiliations:** 1 Department of Chemical and Biological Engineering, Chalmers University of Technology, Gothenburg, Sweden

## Abstract

**Background:**

*Pichia stipitis *and *Pichia pastoris *have long been investigated due to their native abilities to metabolize every sugar from lignocellulose and to modulate methanol consumption, respectively. The latter has been driving the production of several recombinant proteins. As a result, significant advances in their biochemical knowledge, as well as in genetic engineering and fermentation methods have been generated. The release of their genome sequences has allowed systems level research.

**Results:**

In this work, genome-scale metabolic models (GEMs) of *P. stipitis *(iSS884) and *P. pastoris *(iLC915) were reconstructed. iSS884 includes 1332 reactions, 922 metabolites, and 4 compartments. iLC915 contains 1423 reactions, 899 metabolites, and 7 compartments. Compared with the previous GEMs of *P. pastoris*, PpaMBEL1254 and iPP668, iLC915 contains more genes and metabolic functions, as well as improved predictive capabilities. Simulations of physiological responses for the growth of both yeasts on selected carbon sources using iSS884 and iLC915 closely reproduced the experimental data. Additionally, the iSS884 model was used to predict ethanol production from xylose at different oxygen uptake rates. Simulations with iLC915 closely reproduced the effect of oxygen uptake rate on physiological states of *P. pastoris *expressing a recombinant protein. The potential of *P. stipitis *for the conversion of xylose and glucose into ethanol using reactors in series, and of *P. pastoris *to produce recombinant proteins using mixtures of methanol and glycerol or sorbitol are also discussed.

**Conclusions:**

In conclusion the first GEM of *P. stipitis *(iSS884) was reconstructed and validated. The expanded version of the *P. pastoris *GEM, iLC915, is more complete and has improved capabilities over the existing models. Both GEMs are useful frameworks to explore the versatility of these yeasts and to capitalize on their biotechnological potentials.

## Background

*Pichia stipitis *possesses the highest native ability of any yeast to metabolize xylose [1], and is therefore a key candidate for ethanol production from biomass, as well as for engineering xylose metabolism in *Saccharomyces cerevisiae *[2-4]. Xylose consumption requires two additional reactions that are catalyzed by xylose reductase (XYL1, Xyl1p) and xylitol dehydrogenase (XYL2, Xyl2p). Cofactor requirements for these reactions affect the oxygen demand of cells [4]. Xyl1p has a higher affinity for NADPH whereas Xyl2p prefers NAD^+^, hence the formed NADH cannot be properly recycled at oxygen limited conditions [4]. Therefore, the efficient conversion of xylose to ethanol or biomass occurs under defined aerobic conditions. *P. stipitis *produces ethanol at yields close to the maximum at low oxygen transfer rates (~2 mM h^-1^), but xylose uptake rate is only half of the maximum attained under fully respiratory conditions [5]. Attempts to increase the rate of xylose consumption under oxygen-limited conditions have been only partially successful, since the engineered cells were unable to use xylose alone or had reduced ethanol production [6,7]. Due to its advantages in the production of ethanol under anaerobic conditions, while resisting very high concentrations of ethanol, there has been much focus on converting *S. cerevisiae *into a xylose fermenter. However, the expression of target genes from *P. stipitis *into *S. cerevisiae *has not been quite successful due to the difficulties in regulating redox balances during xylose consumption and ethanol conversion [3,4]. Furthermore, the production of ethanol is differently regulated in the two yeasts. Whereas *S. cerevisiae *can produce ethanol via aerobic fermentation (the Crabtree effect) or anaerobically, especially in the presence of high concentrations of glucose [8], *P. stipitis *does it mainly in response to oxygen limitations (Pasteur effect) [5,8,9].

*Pichia pastoris *belongs to a small group of microorganisms capable of catabolizing methanol and fatty acids (e.g. oleic acid) as the sole carbon and energy source [10,11]. It can up-regulate the expression of crucial genes (e.g. alcohol oxidase, *AOX; *and the multifunctional beta-oxidation protein, *FOX2*), and multiply peroxisomes when it is growing on such carbon sources [11,12]. *P. pastoris *has therefore been used extensively for the production of recombinant proteins using the *AOX *gene promoter, as well as a model for studying peroxisome proliferation [11,13]. Consequently, a number of genetic tools and cultivation methods have been developed [13]. Cultivation techniques include fed-batch culture with glycerol, followed by induction with methanol alone, or in combination with glycerol or sorbitol which has shown to be useful for increasing the production of recombinant proteins [14-16]. The success of engineering the glycosylation pathway of *P. pastoris *to produce sialylated glycoproteins has increased the expectations for its use to produce pharmaceutically relevant proteins[17], as well as to transfer the glycosylation technology into *S. cerevisiae *[18].

With the availability of the complete genome sequences for *P. stipitis and P. pastoris *[19,20], there is the opportunity to study, at the system level, the native potentials of these yeasts. Genome-scale metabolic models (GEMs) can be used to support such a task since they can be used to assess metabolic capabilities of cells, to analyze metabolites connectivity and pathway redundancy, or for comparing metabolic capabilities between closely related species [21]. GEMs can also be used to predict genotypic-phenotypic relationships [22], and for the identification of metabolic engineering targets [23]. Moreover, by incorporating 'omics' data and *in silico *methods, GEMs can act as scaffolds for the design of optimal metabolic fluxes [24-26], or to evaluate the correlation between gene expression and metabolic changes in response to environmental perturbations [22,27,28].

Thus far, two GEMs of *P. pastoris *have been published [29,30]. However, neither one incorporates and evaluates methanol metabolism and protein production together, which are among the most important features of this yeast. We also show that our model better predicts the growth phenotype on methanol alone or in combination with glycerol. In this manuscript we report an extensive GEM for *P. pastoris *(iLC915), together with the first GEM for *P. stipitis *(iSS884). Both GEMs correctly represented available experimental phenotypes. The iSS884 model, for example, can predict xylose consumption and distribution into biomass and ethanol using different oxygen up-take rates. The iLC915 model can closely simulate the physiological differences of *P. pastoris *growing at different carbon sources alone (including oleic acid and methanol), as well as methanol mixed with glycerol or sorbitol. iLC915 also includes reactions for the production of a model recombinant protein, for which the production under different oxygen uptake rates compared well with experimental results. The use of such networks on the design of fermentation technologies is also discussed.

## Methods

### Reconstruction of the models

**Figure 1:**
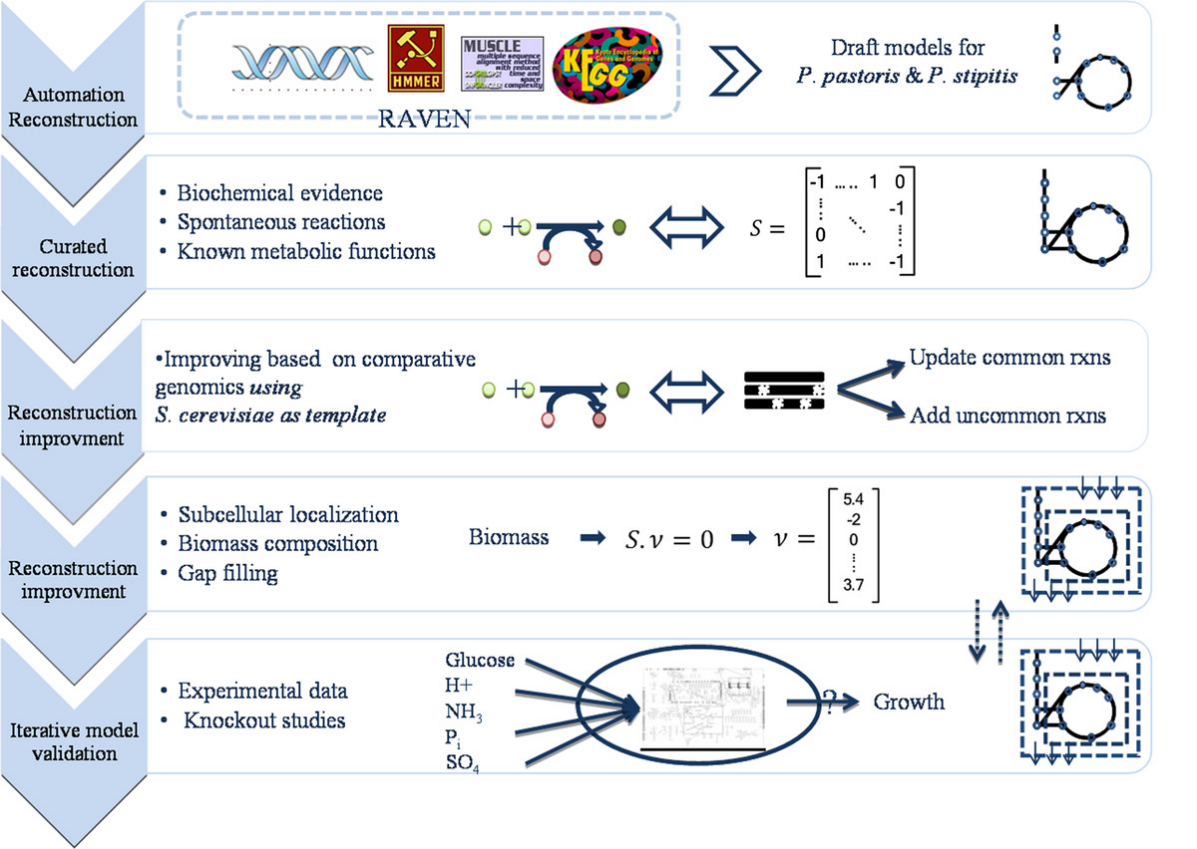
**General steps for the reconstruction of genome-scale metabolic models of *P. stipitis *and *P. pastoris***. Draft models were automatically generated by the RAVEN Toolbox, which also included the primary subcellular localization and transport reactions. Manual curation work was then performed based on known metabolic functions. Gene annotation in the curated model was improved by using functional genomics tools in the third step. The subcellular localization was improved in the fourth step, where the biomass reaction was also added. In this step, the ability of the model to produce all the components to synthesize a cell was checked, and this involved gap filling. In the last step, results from simulations were compared with experimental data. Knockout studies were also done in order to study model robustness.

Reconstruction of the GEMs was comprised by a semi-automatic approach enriched with functional analysis and extensive manual curation based on available literature. Figure 1 depicts an overview of the process. The RAVEN Toolbox was used to generate draft models (manuscript under review). This approach uses a set of reference GEMs of closely related species and infers reactions by means of protein homology. In parallel to this method, the RAVEN Toolbox also uses the KEGG database to infer reactions that may be missing or incorrect in the template models. Draft models were constructed using the genome sequences of *P. stipitis *(CBS 6054) [20], and *P. pastoris *(GS 115) [19]. The iIN800 GEM of *S. cerevisiae *was used as reference framework because of its accurate annotation of fatty acid metabolism, and the extensive information about metabolites and genes, which allowed for a convenient comparison with the KEGG database.

BLASTp (ncbi-blast software ver. 2.2.24) was used to identify homologous proteins among the three yeast species. Protein homologs were identified based on stringent cutoff values (E-values < 10^-40^), and on the score to sequence length ratio according to David et al. (2008) [31]. KEGG Ontology (KO) identifiers were also used to additionally infer reactions which could not be found in *S. cerevisiae *from the genome sequences of the two *Pichia *species following the RAVEN Toolbox pipeline. Finally, the metabolic network of *S. cerevisiae *iIN800 was used to map genes from *P. pastoris *and *P. stipitis *having homologs in *S. cerevisiae*.

Subcellular compartmentalization of reactions was determined using the F-LocA (Fully-connected Localization Assignment), which is part of the RAVEN Toolbox. F-LocA incorporates subcellular localization predictors (CELLO and WoLFPSORT) [32], together with a constraint on network connectivity. Reactions without associated genes were compartmentalized according to biochemical evidence when available. It is important to note that these automated approaches were only used as an aid in the reconstructions, and that biochemical and physiological evidence was always used to validate reaction localizations and gene associations. This was of particular importance in the peroxisomal metabolism where the predictive capability is lower due to the low quality of data from subcellular localization predictors (e.g. CELLO predicts that AOX is in the cytosol, but it is in the peroxisome). In cases where information about *P. pastoris *or *P. stipitis *was lacking, data from other closely related yeasts was used instead (e.g. *S. cerevisiae, Hansenula polymorpha, Candida tropicalis*, *C. shehatea*, and *C. boidinii *[33,34]). Both GEMs are available in the BioMet Toolbox [http://www.sysbio.se/BioMet/ - will be uploaded upon acceptance of paper].

### Biomass reaction and energetic considerations

Biomass reactions were assembled from the macromolecular components (i.e. carbohydrates, proteins, lipids, DNA and RNA). The contribution of each component to biomass (g gDW^-1^), and the appropriate coefficients for every building block present in each macromolecule (mmol g^-1 ^of macromolecule) were calculated based on compositional analysis reports available for both yeast species [19,20,35-38]. These calculations are available in the Microsoft Excel model files.

Production of a model heterologous protein was only considered in the GEM of *P. pastoris *due to its use in recombinant protein production. This protein was the human monoclonal antibody 3H6 Fab fragment (FAB), since there are some experimental reports using it [39,40]. Accordingly, four reactions were included to polymerize nucleotides, ribonucleotides and amino acids separately, as well as for assembling them (Additional file 1). The amino acid composition of FAB was computed from the primary structure submitted to the National Center for Biotechnology Information (NCBI). Since the nucleotide and ribonucleotide sequences have not been reported, these were obtained from the amino acid sequence using *P. pastoris *codon usage reported by De Shutter et al. (2009) [19].

There are two primary ways for a cell to use the generated ATP. The ATP required for biomass synthesis (i.e. precursor biosynthesis and polymerization), and that used for cell maintenance. Both are added to calculate the total amount of energy required for a cell to grow, as it is shown in the following equation:

(1) $$r_{ATP}=Y_{XATP}•\mu +m_{ATP}$$

In equation (1), r_ATP _specifies the total amount of ATP being utilized, Y_xATP _corresponds to the energy utilized for biomass synthesis, μ is the specific growth rate, and m_ATP _is the ATP used for maintenance. The value of Y_xATP _was calculated in two steps according to the procedure previously described [22,41]. First, the amount of ATP demanded for polymerization of cell macromolecules was computed according to Verduyn et al. (1991) [42]. The calculated value is then adjusted by incorporating a scaling factor to fit the predicted slope of the dilution rate vs. glucose-uptake-rate (*μ*/r_glc _= Y_sx _with experimental data [41].

### Constraints-based flux analysis and simulations

Flux balance analysis was used in the simulations with the reconstructed genome scale metabolic models [43,44]. This approach requires a pseudo-steady state to balance metabolite concentrations (*X_i_*). The relation between metabolites' coefficients in the stoichiometric matrix (*S_ij_*) and fluxes through reactions (*v_j_*) is represented in equation (2).

(2) $${S_{i}}_{j}•V_{j}=\frac{dX_{i}}{dt}=0$$

After each simulation an additional flux variability analysis was performed to ensure that the reported fluxes represent the only optimal solution for the resulting objective function value.

### *In silico* prediction of carbon utilization

A general overview of the physiological states through utilization of different carbon sources was performed by feeding 1 C-mol gDW^-1 ^h^-1 ^of each carbon source (e.g. glucose, methanol, xylose, and oleic acid among others), and maximizing for cell growth. Experimental values for specific consumptions were fixed when it was required to predict experimental data. A minimal media was used for all simulations, i.e. reactions for consuming ammonia, sulfate and phosphate remained open during simulations. In simulations with dual carbon sources (e.g. methanol-glycerol or methanol-sorbitol), the upper boundaries of their respective transport reactions were constrained to the experimental fluxes.

### *In silico* production of a recombinant protein

FAB production was evaluated using glucose as carbon source and ammonia as nitrogen source. The objective of these simulations was to assess the effect of oxygen uptake rate and FAB production on the physiological state of cells. This state can depend on the availability of glucose and oxygen, as well as on the metabolic over-load exerted by the transcription, translation and post-translational processing of the recombinant protein. The amount of FAB that cells should synthesize was fixed, since the production of a recombinant protein is subjected to a number of posttranslational processing and regulatory functions, not included in the GEM. The fluxes through the reactions to produce biomass, ethanol and CO_2_, as well as the consumption of glucose, were then quantified and compared against experimental data. Another set of simulations were carried out to evaluate the maximum production of FAB using different carbon sources separately or in combination. In this case, the reaction exporting FAB was used as the objective function and the maximum production was attained.

### *In silico* prediction of the effect of oxygen uptake rate on xylose and glucose fermentation

*In silico *evaluation of the effect of oxygen uptake rate on the production of ethanol and biomass using glucose or xylose was evaluated with the GEM of *P. stipitis*. The oxygen uptake rate (r_O2_) was used as the limiting parameter in simulated continuous cultures with xylose as sole carbon source (note that r_O2 _was fixed as the GEM cannot predict that value). Experimental values of r_O2 _reported by Skoog et al. (1986) [5] were used to fix the upper limits. Simulations were then carried out using the specific production rate of biomass as the objective function. With the results obtained from this evaluation, other simulations were also carried out using a mixture of glucose and xylose at concentrations found in hydrolysates of lignocellulose. These computations were performed in three steps representing the use of continuous reactors in series reported by Grootjen et al. (1991) [45]. In the first reactor a mixture of 40 g/L of glucose and 10 g/L of xylose was fed. Glucose uptake rate was calculated from experimental data. At this step, the production of biomass was favored by using a high r_O2 _value. The stream from this reactor was used to feed the second one. Here, the glucose was completely depleted and xylose started to be consumed at a rate calculated from the experimental data. In this simulation the oxygen uptake rate was decreased to favor the production of ethanol. This strategy was also used in the third reactor where xylose was mainly converted to ethanol.

### *In silico* analysis of reaction essentiality

Reactions deletion in GEMs can be applied by constraining the flux through a single reaction, or to a set of reactions associated with one gene, to zero. Flux balance analysis is then performed to predict the maximum growth rate of the *in silico* mutant strain. The preceding procedure was repeated for all reactions in the network. Percentages of the mutants growth (*Pmw*) with respect to the wild type were calculated using fluxes through the biomass reaction (*Pmw *= ν*_X-mutant_*/ν*_X-wild _*100), and three different labels were assigned. Reactions were classified as essential (E), non-essential (NE), and partially essential (PE). Essential reactions are those where their deletion caused a non-growing phenotype in the *in silico* mutant, getting a *Pmw *of zero. Non-essential reactions have no effect on the growth rate upon deletion. Partially-essential reactions had *Pmw *values between of 0.01-to-0.99, hence a diminished growth rate was seen upon their deletion.

## Results

### Characteristics of the genome-scale metabolic models

GEMs for *P. pastoris*, named iLC915, and *P. stipitis*, named iSS884, are provided in Microsoft Excel, SBML and BioOpt formats and are available in the BioMet Toolbox http://www.sysbio.se/BioMet.

Table 1 gives a summary of the GEMs' main features and how they compare with other models, including the fully compartmentalized consensus model iMM904 of *S. cerevisiae *[46]. The iSS884 model includes 1332 reactions and 922 unique metabolites compartmentalized into four subcellular locations. The number of annotated genes (884) constitutes 15.2% of the total open reading frames (ORFs) found in the genome. The iLC915 contains 1423 reactions and 899 unique metabolites compartmentalized into seven subcellular locations. In total, 915 ORFs were annotated. Comparing with the previous models (PpaMBEL1254 [47] and iPP668 [48]), iLC915 contains more annotated genes (915 compared with 540 and 668, respectively). The number of reactions distributed in the compartments is comparable with the previous GEMs of *P. pastoris *and the iMM904.

**Table 1:** Characteristics of the reconstructed metabolic models iLC915 and iSS884 of *Pichia pastoris *and *stipitis*, in comparison with the *S. cerevisiae *model iIN800 and iMM904

Characteristics	*P. stipitis*	*P. pastoris*			*S. cerevisiae*	
	
	iSS884	iLC915	PpaMBEL1254	iPP668	iMM904	iIN800
Reactions	1332	1423	1202	1354	1312	1446
Cytoplasm	824	790	604	623	635	906
Mitochondria	207	205	155	163	180	161
Peroxisome	60	64	66	66	63	
Vacuole		12	6	3	3	
ER		34	7	15	17	
Golgi		4	8	4	2	
Nucleus			17	16	18	
Extracellular			11	12	7	
Transport	239	314	328	452	387	379
Metabolites	920	899	1147	1177	1168	1013
Gene coverage (%)	15.1	17.2	9.9	12.6	13.7	12.11
Coding genes	5841	5313	5313	5313	6607	6607

Characteristics of the previous GEMs of *P. pastoris *are also included to compare with iLC915

**Figure 2:**
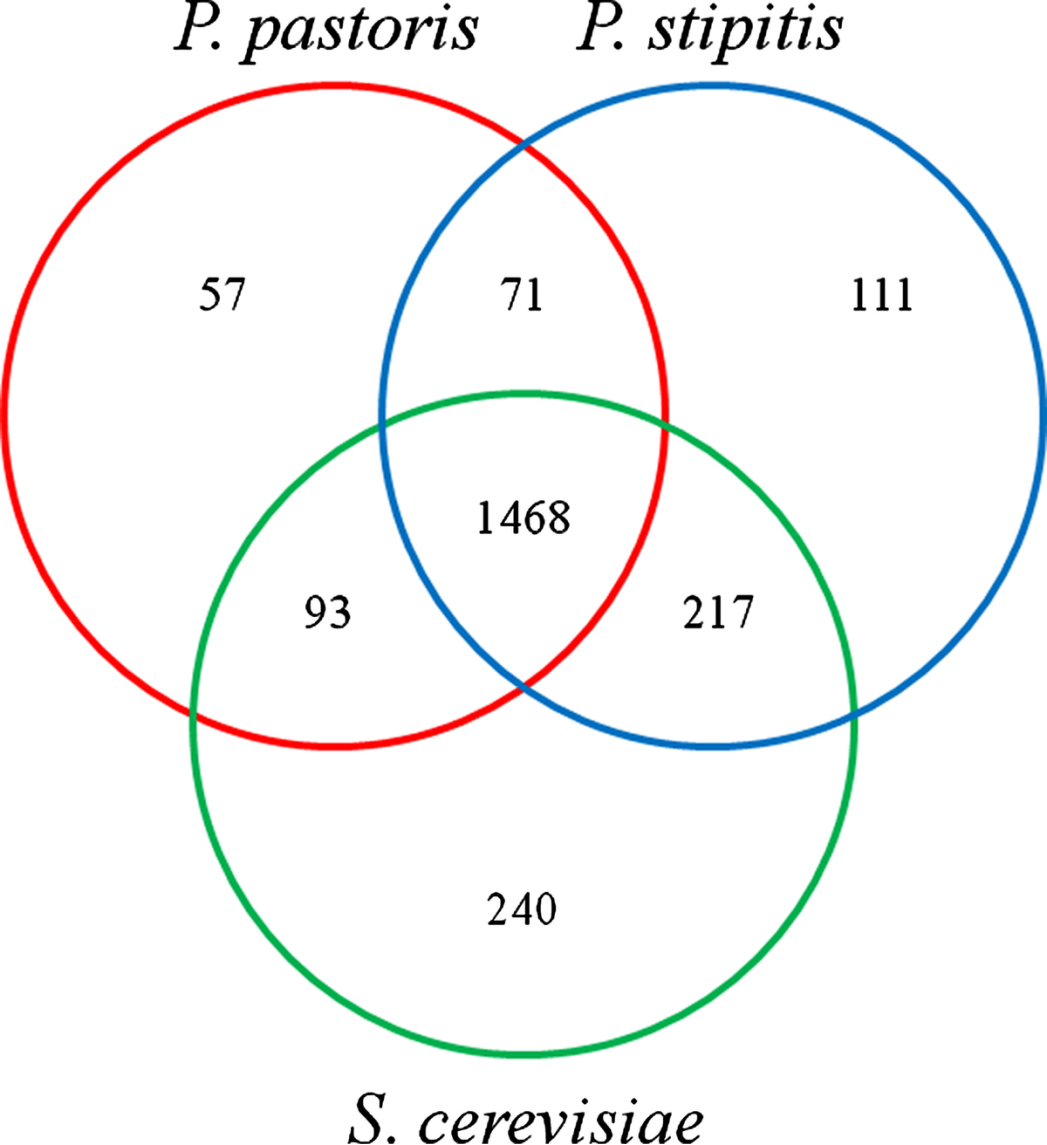
**Comparative genomics based on gene orthologs from the three yeast species, S*. cerevisiae, P. stipitis *and *P. pastoris***. KO identifiers from KEGG were used for this analysis.

Gene orthology analysis using the KEGG orthology (KO) system was performed to improve GEMs reconstruction (Additional file 2). Such an analysis was also valuable to compare the three yeast species (*P. stipitis*, *P. pastoris*, and *S. cerevisiae*) by their GEMs. In total, 1468 genes orthologs are common (Figure 2), of which 522 were annotated in the GEMs (i.e. central, amino acids and nucleotides metabolic reactions). The remaining 946 are associated with cellular processes, as well as genetic expression and regulation, which are not included in the GEMs. In comparison to iIN800 and iSS884, the iLC915 model contains around one hundred more reactions mainly used for posttranslational protein processing and degradation (these are mainly localized into the vacuole, ER and Golgi). 71 genes orthologs are shared solely between *P. pastoris *and *P. stipitis*, of which 16 are associated to the NADH dehydrogenases family (Complex I, also called NADH:ubiquinone oxidoreductase) neither found in *S. cerevisiae *[49], nor in the previous GEMs of *P. pastoris *[47,48]. The substitute oxidases PAS_chr3_0408 and AOX_PICST from *P. pastoris *and *P. stipitis *were not detected during this analysis. These genes were also not included in the previous models for *P. pastoris *[47,48]. However, they have been shown to have an important role in the respiratory metabolism [50,51]. Most of genes orthologs only found for *P. pastoris *and *S. cerevisiae *were not included in the GEMs since they encode functions of cellular and genetic information processing. A higher number of shared genes orthologs were found among *P. stipitis *and *S. cerevisiae *(217). Genes, whose proteins are required to metabolize xylose and arabinose, were annotated in iSS884 and iIN800. There are also some hydrogenases, reductases and phosphatases without gene association in *P. pastoris *(Additional file 2). Genes encoding alcohol oxidases (AOX) and formaldehyde dehydrogenase (FALD), required for methanol metabolism, were found neither in *P. stipitis *nor in S*. cerevisiae*. However, these yeasts have genes that code for the remaining four genes of methanol catabolism (S-formylglutathione hydrolase, formate dehydrogenase, catalase, and dihydroxyacetone synthase). Additional file 2 is available for further information.

### *In silico *prediction of reaction essentiality

*In silico *prediction of reaction essentiality was performed. Results have been distributed in three categories: essential, partially-essential, and non-essential (see materials and methods section). In total, 1417, 1328, and 1361 unique reactions were deleted individually from iLC915, iSS884 and iIN800 GEMs, respectively. Out of these deleted reactions, 173 in iLC915, 155 in iSS884 and 193 in iIN800 were found to be essential for growth in minimal media with glucose. Reactions which only caused growth retardation (partially-essential) accounted for 85 in iLC915 and 58 in iSS884 and iIN800.

**Figure 3:**
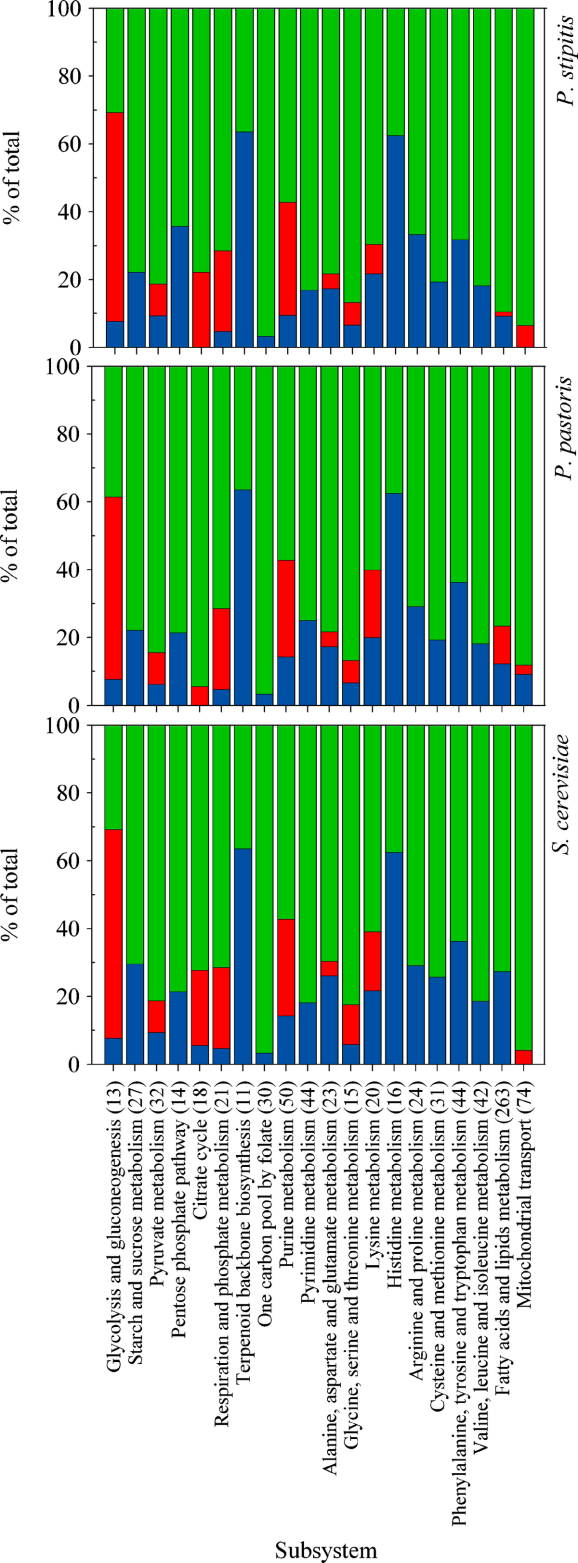
***In silico *analysis of reaction essentiality between *P. pastoris, P. stipitis*, and *S. cerevisiae *cultivated in minimal media with glucose**. Reactions from GEMs of the three yeasts were deleted one by one and growth was maintained as the objective function. Reactions were classified according to the resulting phenotype; essential is where cells stop to grow after the deletion (red); partially-essential means a reduction in growth rate (blue); and non-essential when no effect on growth was seen (green). Reactions were arrangement in pathways for a better comparison.

Deleted reactions for the three models were grouped into different metabolic pathways (Figure 3 and Additional file 3). Reactions deleted in amino acid synthesis grouped most of the essential genes, with the highest score for essential-reactions in the metabolism of histidine. Biosynthesis of the backbone for terpenoids accumulated 6 lethal reactions comprising 60% of the reactions included in this pathway. The highest amount of partially-essential reactions was distributed in the central carbon metabolism. The iIN800 model of *S. cerevisiae *accumulated a higher number of lethal phenotypes in reactions located in the lipids network (25%, compared to 10% in the other yeasts), which is likely due to the fact that the iIN800 comprises more details on lipids composition in the biomass equation [52].

### *In silico *evaluation of carbon utilization

**Figure 4:**
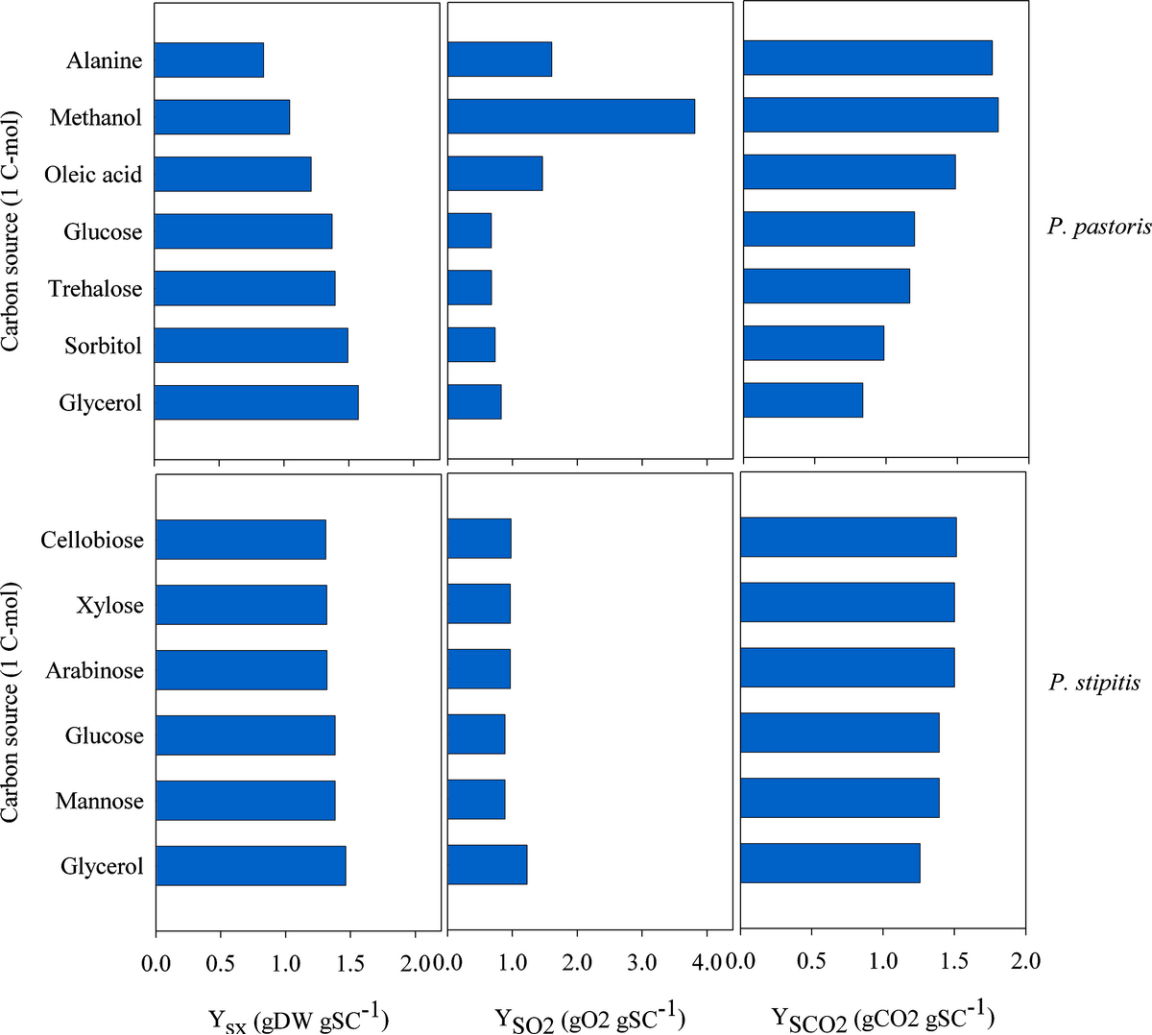
***in silico *evaluations of yields during the cultivation of *P. pastoris *and *P. stipitis *on different carbon sources**. Predicted results are reported as follow, from left to the right: biomass yield (Y_SX_), O_2 _yield (Y_SO2_), and CO_2 _yield (Y_SCO2_).

*In silico *evaluation of carbon assimilation using the iLC915 and iSS884 GEMs was carried out in simulated chemostats with minimal media. With iSS884, glycerol was better assimilated (Figure 4), followed by glucose and mannose .Xylose, arabinose and cellobiose showed the lowest conversion yields. The maximum conversion of carbon to biomass in the *P. pastoris *model iLC915 was computed for glycerol and sorbitol, followed by glucose and trehalose. Lower values were obtained with oleic acid, alanine and methanol. Simulations also reproduced the higher oxygen requirements to oxidize methanol, alanine and oleic acid, which is the first step for their metabolism.

Energy requirements for biomass synthesis strongly depend on the nature of the carbon source [53]. The total amount of ATP required to produce one gram of biomass (Y_xATP_) and the efficiency of aerobic respiration measured as P/O ratio were calculated for each carbon source. With glucose, glycerol, sorbitol, or trehalose, the computed values for Y_xATP _using the iLC915 GEM were 70.5, 76.5, 73.1, and 74.2 mmolATP gDW^-1^, respectively. Even when the biomass yield with alanine was low compared with the other carbon sources, a comparable result of the Y_xATP _was calculated (72.3 mmolATP gDW^-1^). The highest energy requirements for growth were estimated with methanol and oleic acid (152.5 and 95.8 mmolATP gDW^-1^). Mixtures of glycerol with methanol at decreasing ratios (i.e. 4.33, 1.44, and 0.70 grams of glycerol per gram of methanol) showed increasing Y_xATP _values (80, 87, and 94 mmolATP gDW^-1^, respectively), and reducing biomass yields (Y_sx_) accordingly (0.51, 0.48, and 0.45 gDW g^-1^). With the iSS884 model of *P. stipitis*, ATP required for biomass formation was similar for some carbon sources (glycerol, 95.5 mmolATP gDW^-1^; glucose and mannose, 82.2 mmolATP gDW^-1^). Xylose, arabinose, and cellobiose assimilation needed 87.5, 87.4 and 86.0 mmolATP gDW^-1^, respectively.

### Comparison between *in silico *phenotypic predictions and experimental data

**Figure 5:**
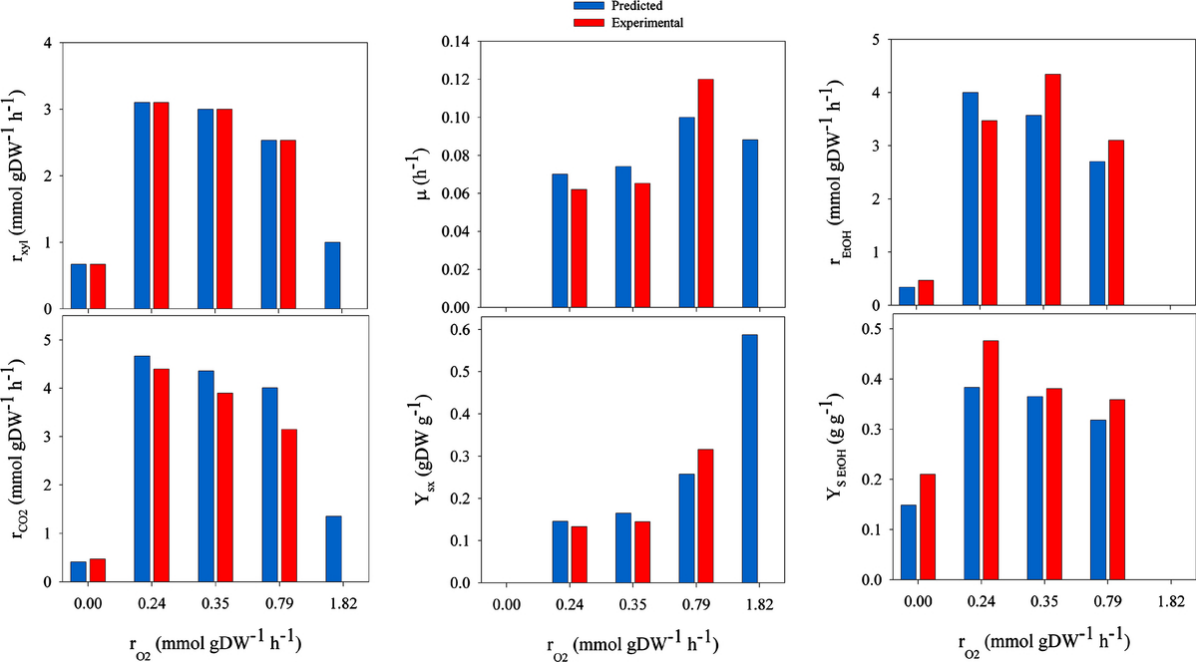
**Comparison between predicted and experimental results for the production of biomass and ethanol by *P. stipitis *using xylose as a substrate**. Comparisons are based on different oxygen uptake rates (r_O2_). Yields of biomass and ethanol (Y_sx _and Y_xEtOH_), specific rates of ethanol and CO_2 _production (r_EtOH _and r_CO2_), and xylose consumption (r_Xyl_) are compared.

Only a few studies on continuous culturing of *P. stipitis *to investigate its physiology have been done [5,54]. Particularly, the oxygen transfer rate is a crucial factor for xylose metabolism that, at defined levels, can maximize the productivity and yield of ethanol. The role of oxygen in xylose fermentation can be explained by the fact that cells have to maintain redox balance, xylose transportation, cell growth or keep mitochondrial function [55]. The iSS884 model was used to evaluate the metabolism of xylose by constraining it to experimental oxygen transfer rates. Results from *in silico *predictions of biomass yields (Y_sx_), dilution rates (μ), ethanol yields (Y_sEtOH_), CO_2 _specific productivities (r_CO2_) and ethanol specific productivities (r_EtOH_) were compared with experimental data. Figure 5 shows that the GEM predicts the correlation of oxygen transfer rate with metabolism, which passed from fermentative to respiratory. These results are in agreement with experimental data [5]. Furthermore, *in silico *simulations predicted the inability of *P. stipitis *to grow in anaerobic conditions, as well as the small amount of ethanol that cells produce *in-vivo*. Xylitol production was not observed neither in the experimental results [5], nor in the *in silico *evaluations (indicating that the regeneration of NAD^+ ^was not limiting).

**Figure 6:**
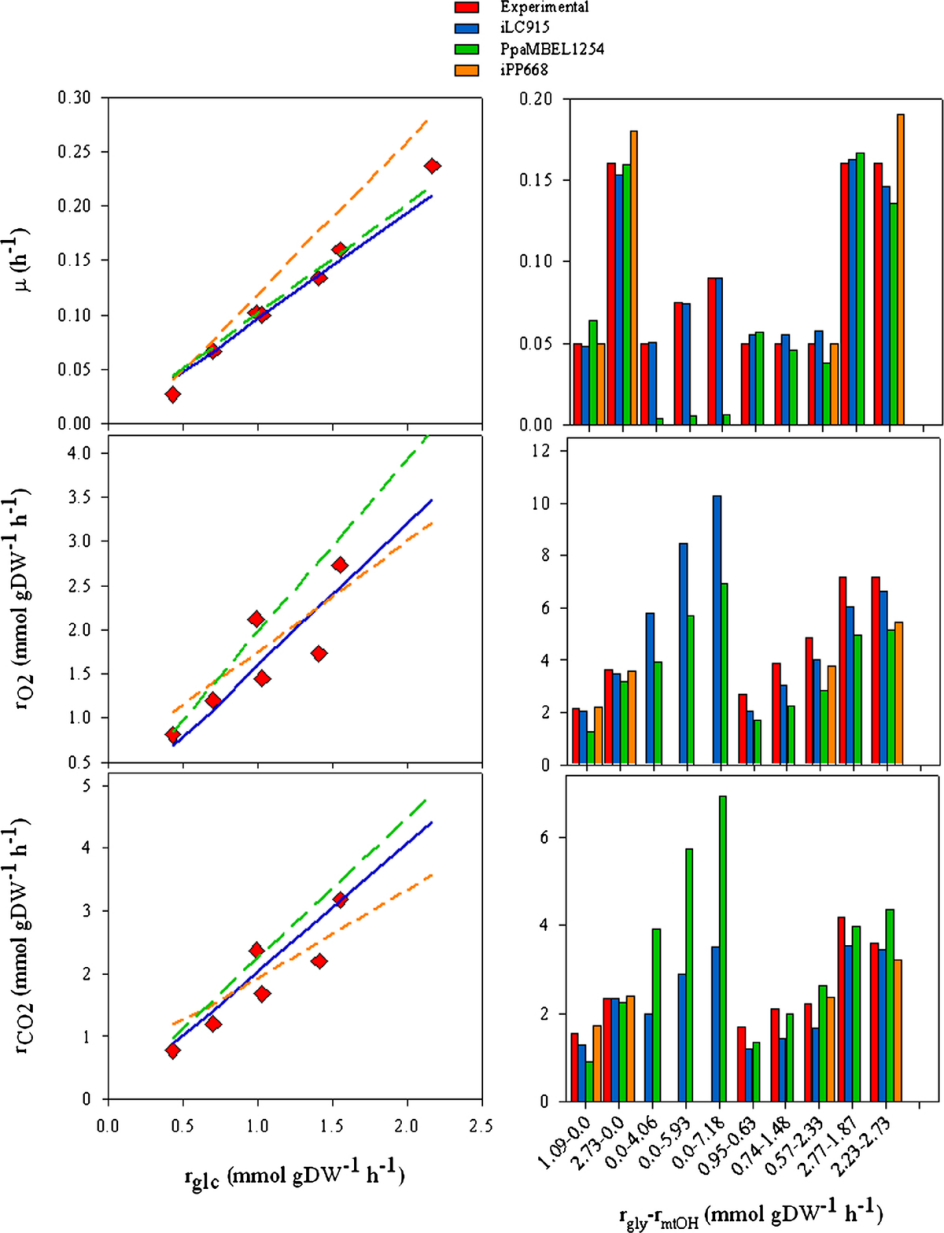
**Comparisons of simulated and experimental values for the growth of *P. pastoris *in glucose, glycerol and methanol, and mixtures of methanol and glycerol**. Values of the specific growth rate (μ), O_2 _uptake rate (r_O2_) and CO_2 _production rate (r_CO2_) from simulations with the three GEMs are compared.

*In silico *physiological predictions with the iLC915 GEM of *P. pastoris *using glucose, methanol, glycerol and a mixture of methanol with glycerol at various dilution rates were also carried out. Predicted values for specific growth rates (μ), oxygen consumptions (r_O2_), and CO_2 _productions (r_CO2_) are shown in the Figure 6. Comparing with PpaMBEL1254, the iLC915 had a similar capacity to predict *P. pastoris *phenotype on glucose. Such predictions were less accurate when using the iPP668 model, which computed higher μ values. This was also the case when the models were used to simulate glycerol metabolism. Results of methanol utilization in the three GEMs were different. While iPP668 predicted higher values of μ, the PpaMBEL1254 computed lower ones (0.0135 and 0.005 h^-1^). This was reflected in the amount of CO_2 _produced by both models (0.91 and 0.392 mmol/gDW^-1^). Computations with iLC915 provided values of 0.0125 h^-1 ^and 0.48 mmol/gDW^-1 ^for μ and r_CO2_. Those values were closer to *P. pastoris *phenotype on this carbon source, as it is shown by comparing with experimental data (Figure 6). Furthermore, the better prediction of *P. pastoris' *physiology with iLC915 resulted in improved estimates when methanol and glycerol were used simultaneously.

### *In silico *simulation of a recombinant protein production using the *P. pastoris *model iLC915

To evaluate the ability of iLC915 for simulating recombinant protein production, we looked at the production of the human monoclonal antibody 3H6 Fab fragment (FAB), which has been experimentally assessed [39,40]. Simulations were carried out in simulated chemostats with glucose at different oxygen uptake rates (Table 2). It should be noted that simulations just included the metabolic adjustments of the biochemical responses according to the metabolic capabilities represented by the GEM constrained with experimental data. Cellular processes like molecular regulation of stress responses and protein quality control are not included.

**Table 2:** *In-silco *prediction of experimental parameters during the FAB production using three different oxygen uptake rates.

Parameter	Case 1 O2 inlet air = 8%		Case 2 O2 inlet air = 11%		Case 3 O2 inlet air = 21%	
	
	Pred	Exp	Pred	Exp	Pred	Exp
Y_XFAB _(mg gDW^-1^)	0.54		0.38		0.22	
r_O2 _(mmol gDW^-1 ^h^-1^)	4.14		2.26		2.23	

Y_SX _(gDW g^-1^)	0.31	0.25	0.43	0.45	0.48	0.47
r_Glc _(mmol gDW^-1 ^h^-1^)	1.81	1.85	1.28	1.27	1.15	0.98
r_CO2 _(mmol gDW^-1 ^h^-1^)	5.11	5.62	2.85	2.57	2.69	2.34
r_EtOH _(mmol gDW^-1 ^h^-1^)	0.71	1.12	0.11	0.17	0.00	0.00

Experimental data were taken from Baumann et al., (2008 and 2010) [39,40]

Table 2 shows a comparison between predicted and experimental data. With constant values of Y_xFAB _and r_O2_, model predictions are very close to the experimental values. The maximum biomass yield was obtained experimentally and *in silico *under fully aerobic conditions (case 1). By decreasing the percentage of oxygen in the inlet air to 11 and 8%, Baumann et al. (2010) [40] observed a 2-fold higher specific oxygen uptake rate. By setting these values to be constant, the simulations were unable to predict the specific rate of glucose consumption and ethanol production (data not shown), as a fully respiratory metabolism is preferred. However, after forcing glycolytic fluxes through the pyruvate node, as it was observed by Baumann et al. (2010)[40], the simulations were capable to predict the flux of glucose necessary to complete carbon requirements for biomass and protein synthesis, as well as to cover energy consumption and ethanol production under such respiro-fermentative conditions.

## Discussion

The genome-scale metabolic models iLC915 and iSS884 of the methylotrophic yeast *P. pastoris *and the xylose fermenting *P. stipitis *were reconstructed and evaluated. Taken together, the results of constraint-based analysis used to evaluate cellular functions of these models were in close agreement with experimental results (Figures 6 and 5, and Table 2). Predicted physiological states of both yeasts in relation to biomass yields computed for different carbon sources of experimental relevance compared well to experimental values compiled by Verduyn (1991)[53]. ATP yields calculated by fitting the reconstructed model to experimentally-determined biomass yields on different carbon sources reproduced the dependence of Y_xATP _on the nature of the carbon source previously observed [42,53]. Such calculations were extended to estimate the physiological benefits of the co-assimilation of glycerol with methanol using the iLC915 model of *P. pastoris *(Y_xATP _decreased as the ratio of glycerol/methanol increased).

**Figure 7:**
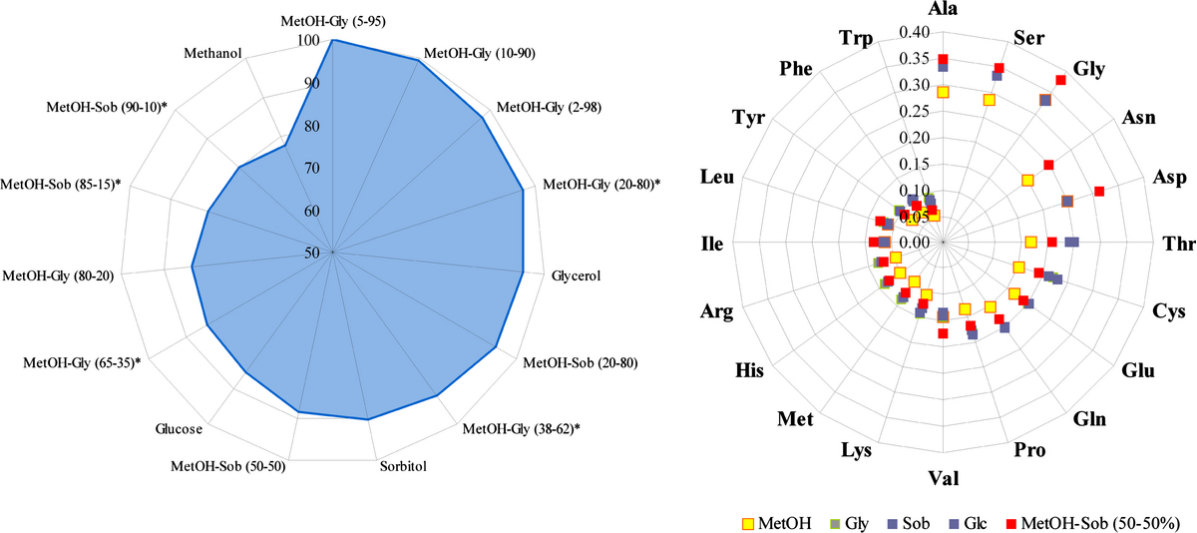
**Comparison between the maximum capacities for amino acids and recombinant protein production with different carbon sources using the iLC915 model of *P. pastoris***. The production of amino acids was evaluated with glucose, glycerol and methanol, as well as with a mixture of methanol with sorbitol, data is expressed in mole of amino acid per carbon mole of substrate. The production of FAB was simulated with glucose, glycerol, sorbitol and methanol, and mixtures of methanol with glycerol or sorbitol. FAB production was used as the objective function. Data is expressed as the percentage of the maximum production, which was obtained with a mixture 5-95% of methanol and glycerol. Asterisks over some mixtures indicate that such combinations were already evaluated in experimental work, and therefore can be used in a production process.

Several non-genetic approaches have been tested in order to optimize the production of recombinant proteins using *P. pastoris *as host. One of them included the use of other well-assimilating carbon sources (e.g. glycerol and sorbitol) together with methanol, which is poorly assimilated and very toxic [14,15]. GEMs can be used to predict the capacity of *P. pastoris *for the synthesis of amino acids and recombinant proteins using methanol alone or in combination (Figure 7). Methanol is the worse carbon source for the synthesis of amino acids (since it is not well assimilated) and recombinant proteins. However, when it is used together with glycerol (e.g. 20-80%), the production increases to levels comparable to those obtained with glycerol alone. Predictions also suggested that some combinations of methanol-glycerol may be better to increase the production of FAB when compared to glycerol, sorbitol or glucose as the sole carbon source. Since simulations cannot predict the effect of such mixtures on the transcription of the *AOX *gene promoter, these findings must be complemented with experimental work.

**Figure 8:**
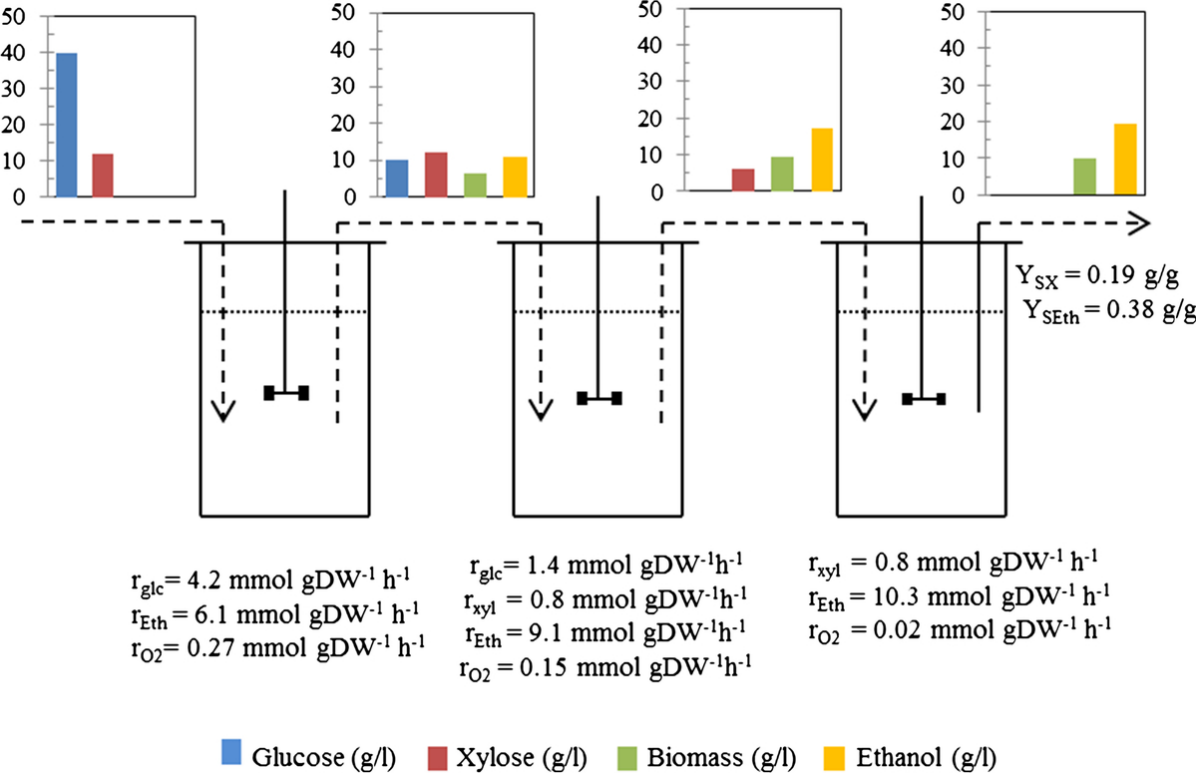
***In silico *conversion of glucose/xylose mixture by iSS886 in three reactors in series**. In the first reactor the glucose is the only substrate for growth, and in the second a mixture of glucose/xylose is used as substrate. In the third reactor only xylose was consumed. The simulations were done based on cell growth as the objective function. The ethanol production rate and specific growth rate are also reported.

*P. stipitis *has been used to produce ethanol from a synthetic media simulating the concentrations of glucose and xylose remaining after the hydrolysis of lignocellulose [45]. The metabolism of glucose exerts catabolic repression. Hence, xylose uptake starts after depletion of glucose. Grootjen et al. (1991)[45], therefore proposed the sequential fermentation of glucose-xylose mixtures using reactor in series. This strategy was reproduced *in silico *using the iSS884 model (Figure 8). Simulations were performed by feeding the media with glucose and xylose in the first *in silico* bioreactor. Here, the glucose is rapidly metabolized to produce biomass and ethanol. The output stream that contains xylose, remaining glucose, ethanol and biomass produced in the first reactor was the input for the second reactor. Herein glucose was completely depleted and almost half of the xylose was converted mainly into ethanol. However, the productivity of ethanol was lower than in the first reactor, since the consumption of glucose occurred faster than xylose. The stream coming out from the second reactor contained only xylose as carbon source. This was fed to the third reactor, where it was converted completely into ethanol. Results from this simulation are in close agreement with the data reported by Grootjen et al. (1991) [45], and shows the potential of the simulations with the iSS884 GEM for the design of strategies to produce ethanol from different combinations of glucose and xylose coming from the hydrolysis of lignocellulose.

By comparing the reconstructed GEMs of both *Pichia *species with *S. cerevisiae*, we found that the maximum capacities for the production of the 20 amino acids and the essentiality of reactions for the three yeasts to grow on glucose are similar (Additional file 4). This can be explained by the large number of shared metabolic reactions, in particular in the central, amino acid, lipid and nucleotide metabolism (Additional file 2). Llorente et al. (2000a and b)[56,57] suggested, based on a comparative analysis of chromosome maps and synteny with *S. cerevisiae*, that yeasts evolution may be driven by a balance between gene duplications and deletions, given change to the existence of orthologous and paralogous genes. Genes coding for proteins involved in basic tasks are quite conserved, meanwhile unnecessary gene copies have been deleted rather than conserved, and hence unusual metabolic functions are almost entirely absent [58]. The latter applied for the particular capabilities of *P. pastoris *and *P. stipitis *to metabolize methanol and xylose as a sole carbon source. The metabolism of xylose is a singular case, since even when *S. cerevisiae *has coding genes to transport and reduce xylose, as well as for the dehydrogenation of xylitol, it cannot grow on xylose as a sole carbon source. This suggests that unusual metabolic traits may emerge not from the use of existing enzymes, but from a larger number of genes and/or regulatory factors found in the native organisms [59]. Metabolic modeling at genome scale is a useful tool to investigate these traits.

## Conclusions

In summary, we presented two high quality GEMs that can be used to gain understanding on the metabolic capabilities of the two yeasts *P. stipitis *and *P. pastoris*. These models also provide a versatile tool for rationale strain improvement, scaffold for data integration and interpretation and evolutionary analysis of yeasts metabolism.

## Competing interests

The authors declare that they have no competing interests..

## Authors' contributions

LC and SS reconstructed the GEMs, designed and performed simulations, and wrote the manuscript. RA and IN assisted with the reconstruction and modeling and reviewed the manuscript. JN supervised the work and assisted in manuscript preparation. All authors read and approved the final manuscript.

## Supplementary Material

Additional file 1 **Composition of the human monoclonal antibody 3H6 Fab fragment (FAB) used as model recombinant protein**. Click here for file

Additional file 2 **Gene orthology analysis among the three yeast species: *Pichia stipitis, Pichia pastoris, and Saccharomyces cerevisiae***. Click here for file

Additional file 3 **Analysis of reaction essentiality predicted by simulations**. Click here for file

Additional file 4 **Maximum capacities for amino acids production among the three yeast species: *Pichia stipitis, Pichia pastoris, and Saccharomyces cerevisiae***. Click here for file
