# Supplementary material for: Genome-scale metabolic reconstructions of Pichia stipitis and Pichia pastoris and in silico evaluation of their potentials
Source: BMC Syst Biol. 2012 Apr 4;6:24. doi: 10.1186/1752-0509-6-24 (PMC3364918; doi:10.1186/1752-0509-6-24)
Supplement: Additional file 4 — Maximum capacities for amino acids production among the three yeast species: Pichia stipitis, Pichia pastoris, and Saccharomyces cerevisiae. [file 1752-0509-6-24-S4.DOC]

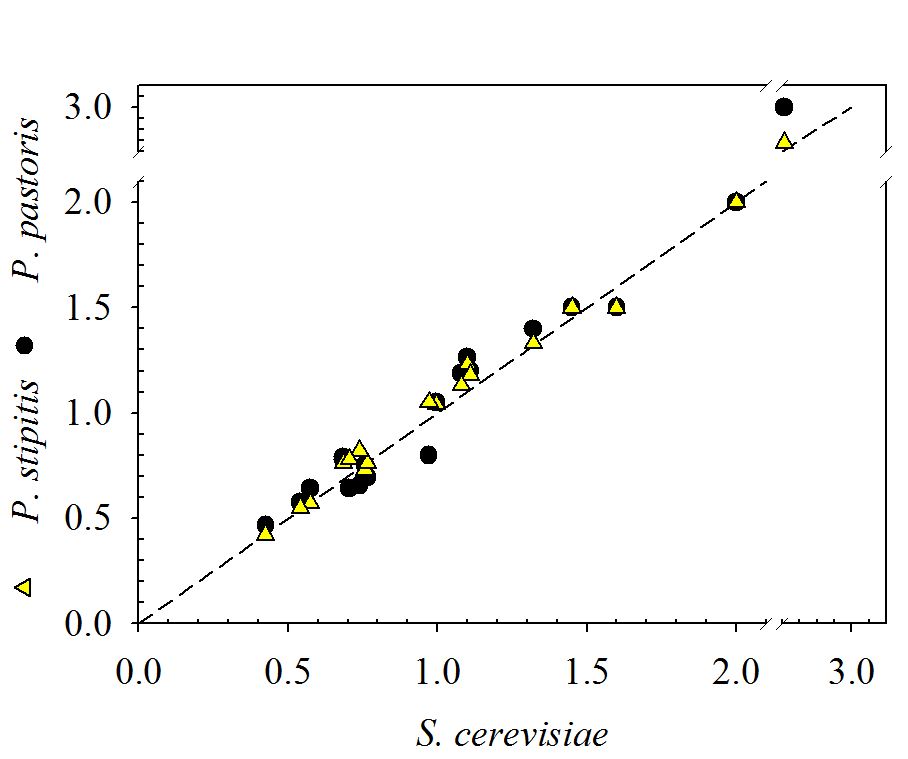


**Maximum capacities for the production of amino acids among the three yeast species predicted by simulations.** Reactions for the synthesis of every amino acid were maintained as objective function and simulations to compute its maximum production were performed in minimal medium with glucose as the carbon source.
